# Anisotropic Elasticity of the Myosin Motor in Muscle

**DOI:** 10.3390/ijms23052566

**Published:** 2022-02-25

**Authors:** Marco Caremani, Massimo Reconditi

**Affiliations:** 1 PhysioLab, Università di Firenze, 50019 Sesto Fiorentino, Italy; marco.caremani@unifi.it; 2 Dipartimento di Biologia, Università di Firenze, 50019 Sesto Fiorentino, Italy; 3 Dipartimento di Medicina Sperimentale e Clinica, Università di Firenze, 50134 Firenze, Italy

**Keywords:** myosin, molecular motors, muscle mechanics, protein elasticity

## Abstract

To define the mechanics and energetics of the myosin motor action in muscles, it is mandatory to know fundamental parameters such as the stiffness and the force of the single myosin motor, and the fraction of motors attached during contraction. These parameters can be defined in situ using sarcomere−level mechanics in single muscle fibers under the assumption that the stiffness of a myosin dimer with both motors attached (as occurs in rigor, when all motors are attached) is twice that of a single motor (as occurs in the isometric contraction). We use a mechanical/structural model to identify the constraints that underpin the stiffness of the myosin dimer with both motors attached to actin. By comparing the results of the model with the data in the literature, we conclude that the two-fold axial stiffness of the dimers with both motors attached is justified by a stiffness of the myosin motor that is anisotropic and higher along the axis of the myofilaments. A lower azimuthal stiffness of the motor plays an important role in the complex architecture of the sarcomere by allowing the motors to attach to actin filaments at different azimuthal angles relative to the thick filament.

## 1. Introduction

The sarcomere is the structural unit of striated muscle (skeletal and cardiac). In each sarcomere (~2–2.5 μm long), the contractile protein myosin and actin are organized in well₋ordered and parallel arrays of filaments. The thick myosin−containing filament, at the center of the sarcomere, overlaps with the thin actin−containing filament originating from the Z−line bounding each end of the sarcomere. On each half of the thick filament, the dimeric molecular motor myosin II is regularly arranged in an array of 147 myosin dimers, for a total of 294 motors. Force and shortening are driven by cyclical interactions of the myosin motor with the thin filament, fueled by the hydrolysis of ATP. In the contracting muscle, the myosin motor (also called the myosin head) attaches to actin, forming a cross−bridge, and undergoes a structural working stroke that drives the thin filament towards the center of the sarcomere. The parallel and series arrangement of the sarcomeres accounts for the macroscopic force and the shortening of the muscle. The arrays of 294 motors work cooperatively in each half−sarcomere (hs, the functional unit of striated muscle), so that in order to define the molecular basis of muscle energetics and efficiency, it is necessary to know either the stiffness and the force of the attached myosin motor (cross−bridge) or the fraction of motors (*f*) attached in an isometric contraction, and how this fraction depends on the load or shortening velocity (for an extensive reference see [1]). The most suitable preparation for measuring those parameters under physiological conditions is the single muscle cell, or fiber, in which the measurements can be made at the level of the half-sarcomere, with nm−μs resolution [2,3,4,5]. *f* can be determined by comparing the compliance of the half−sarcomere (*C*_hs_) during contraction and in rigor, the state reached after the depletion of ATP, with all 294 of the myosin motors in each half-thick filament attached to actin [4,6]. In those studies, it was assumed that the stiffness of a dimer with both motors attached to actin, such as in rigor, is twice that of the single attached motor, as occurs in the contracting muscle [7], given that the compliance of the rod-like link between the motors and the thick filament, the S2 myosin subdomain, is effectively infinitely stiff when a tensile force is applied along its axis [8,9]. This assumption does not consider that when both motors of the same dimer attach to consecutive monomers on the actin filament while retaining their common attachment to S2, they must undergo a distortion that complicates the relationship between the stiffness of the head in rigor and that of a single head attached during active contraction [10]. Here we show, through a mechanical−geometrical model of the two−motor attached dimer, that the constraint of sharing the head−rod junction would increase the apparent axial stiffness of the motors in rigor by at least a factor of two relative to that of the unconstrained motor. There are experimental indications, however, that this is not the case. To reconcile our theoretical analysis with the experimental data, we suggest that the stiffness of an actin−attached motor is not isotropic, and that the lateral, or azimuthal, stiffness is one order of magnitude lower than the axial stiffness.

## 2. Results

### 2.1. The Stiffness of a Constrained Elastic Element

When the elastic deformation of an element with different stiffness values along different directions is subject to a structural constraint, the apparent stiffness, i.e., the ratio between the stress (force) and strain (elongation) in a given direction, is determined also by the geometry of the constraint.

**Figure 1:**
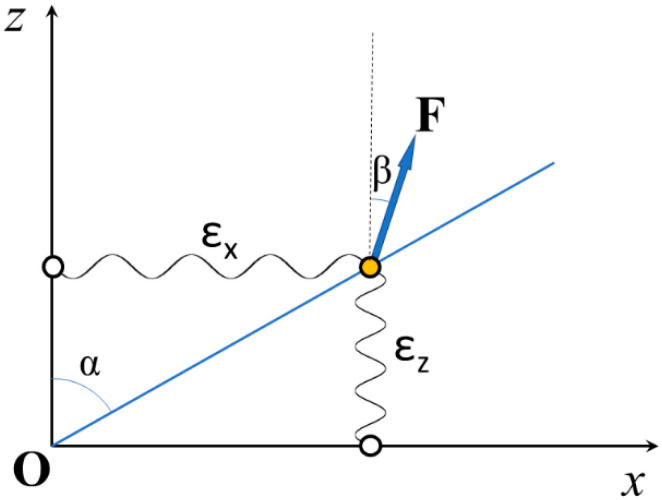
Schematic 2D representation of an elastic element with different stiffness values ε_x_ and ε_z_ along the *x* and *z* directions, respectively. The blue line at angle α with the *z* axis represents the structural constraint for the movement of the tip of the element (orange circle) when force F (blue arrow) is applied.

Figure 1 shows a simple example of a 2D constraint for the deformation of an elastic element. The blue line at angle α with the *z* axis represents the structural constraint for the movement of the tip of the element (orange circle) when force **F** at angle β with the *z* axis is applied. The calculations developed in Appendix A show that the apparent stiffness $\underset{\varepsilon_{z}}{\sim }$ along the *z* direction is given by:

(1) $$\underset{\varepsilon_{z}}{\sim }=\cos(\beta )\cdot \{\varepsilon_{x}\cdot \frac{\sin^{2}(\alpha )}{\cos(\alpha )\cdot \cos(\alpha -\beta )}+\varepsilon_{z}\cdot \frac{\cos(\alpha )}{\cos(\alpha -\beta )}\}$$

where ε_x_ and ε_z_ are the stiffness of the unconstrained element along the *x* and *z* directions, respectively.

When the force **F** is applied along the *z* direction, i.e., β = 0, then:

(2) $$\underset{\varepsilon_{z}}{\sim }=\varepsilon_{x}\cdot \tan^{2}(\alpha )+\varepsilon_{z}$$

These results show that the apparent stiffness of a constrained elastic element may be higher than it would be without the constraint. If, for example, the stiffness were the same, ε, in both directions, i.e., ε_x_ = ε_z_ = ε, then Equation (2) would become:

(3) $$\underset{\varepsilon_{z}}{\sim }=\varepsilon \cdot \left(\tan^{2}\left(\alpha \right)+1\right)$$

with tan^2^(α) varying between 0 and ∞, and $\underset{\varepsilon_{z}}{\sim }$ between ε and ∞.

### 2.2. The Structural Constraint for the Two Motors Attachment in Rigor

**Figure A1:**
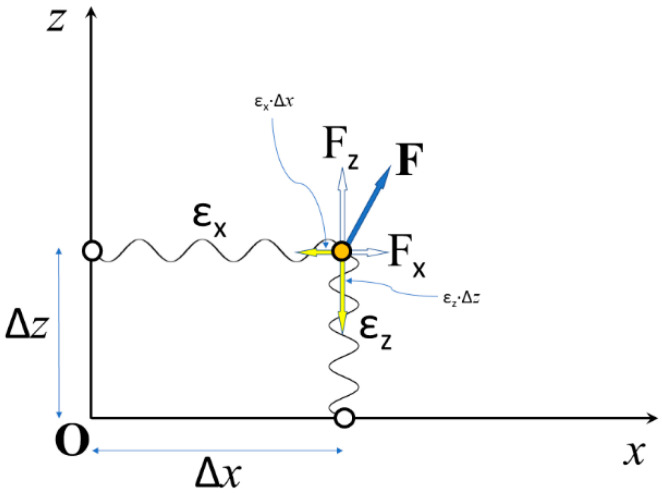
Schematic 2D representation of an elastic element with different stiffness values of ε_x_ and ε_z_ along the *x* and z directions, respectively. An external force **F** (blue arrow) applied to the tip of the elastic element (orange circle) is equilibrated by the strains Δ*x* and Δ*z,* eliciting the restoring forces (yellow arrows) that balance the component of F along the *x* (F_x_) and *z* (F_z_) directions, respectively (white arrows).

When a myosin dimer attaches to the actin filament via a single motor, there are no constraints for the displacement of the tips of the two myosin motors that, when an external force is applied, would respond with a strain in a direction that depends on both its stiffness and the intensity and direction of the applied force (Appendix A and Figure A1).

When both motors are attached to two consecutive monomers of the same strand of the actin helix, their shared attachment at Lys843 to the S2 rod of the two S1 fragments sets a structural constraint for their movement. Here, we have assumed that each S1 behaves as a rigid body that is allowed to rotate around the Cys707 residue, and with its compliance residing in the hinge itself. It must be noted that Cys707 is indicated by crystallographic models as the site around which the myosin motor undergoes the structural change responsible for the working stroke [11,12], and thus it is considered as the beginning of the lever arm. However, Seebohm and colleagues [13] have suggested that the compliance of S1 resides mostly in the non-conserved residues 719 and 723 (Arg719 and Arg723 in the cardiac β−isoform studied there), not far from Cys707. For simplicity, and with negligible effects on the results, we have considered Cys707 as the site for both lever arm rotation and motor compliance [9,14,15].

**Figure 2:**
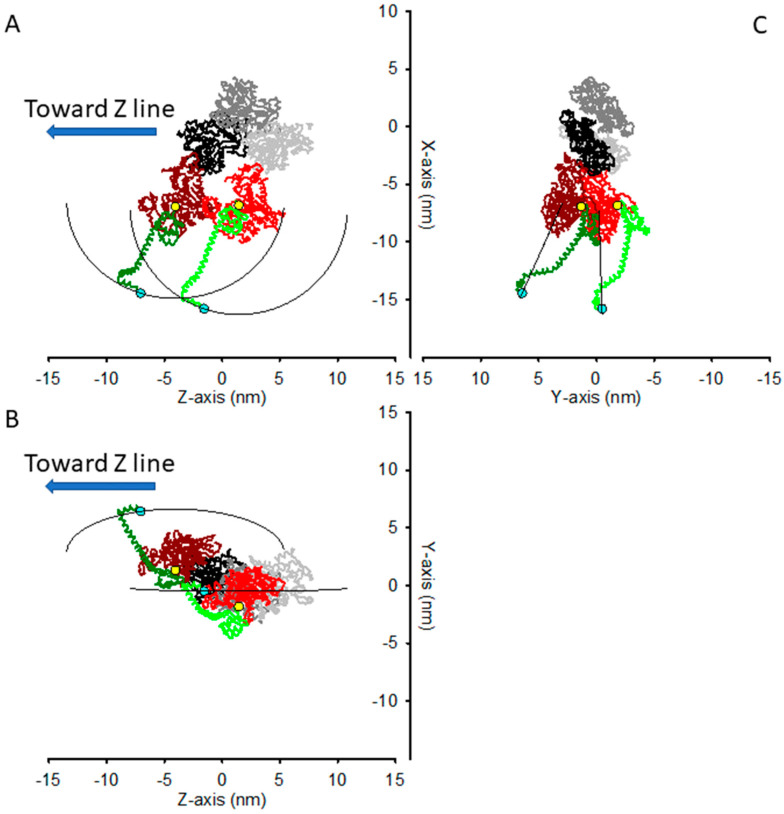
Structural model for two myosin motors, in the rigor conformation, attached to two consecutives actin monomers on the same strand of the actin helix. Gray and black: actin monomers; red and brown: myosin motors’ catalytic domain; green (light and dark): lever arm; yellow circles: pivoting points on Cys707; cyan circles: tip of the lever arm at Lys843. The Regulatory (RLC) and Essential (ELC) Light Chains wrapped around the lever arm are not shown to better visualize the lever arm’s orientation. Atomic coordinates from [16]. (**A**,**B**) Lateral views across the actin filament axis (*Z* axis). (**C**) View along the direction of the filament axis. The thin black lines represent the trajectories of the res843s when the lever arms rotate on planes containing the *Z* axis (axial movement).

If the two motors were attached to consecutive actin monomers along the same strand of the helix in the rigor conformation, as determined in [16], without the constraint of sharing the S2 attachment, then the distance between the two Lys843 would be 8.94 nm (Figure 2). This would be the case, for example, for the so called S1−decorated actin filament [17].

**Figure 3:**
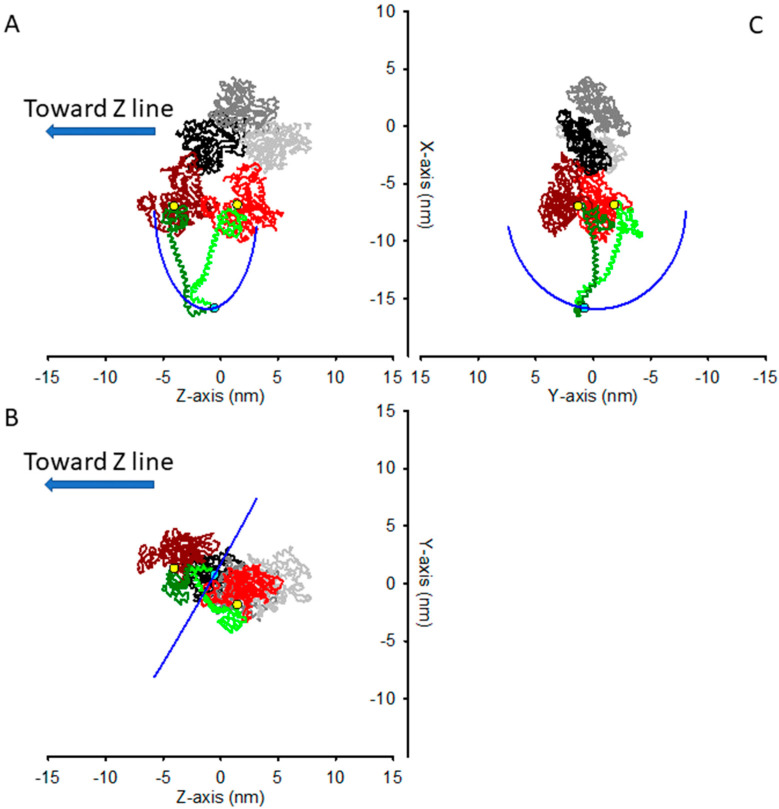
As in Figure 2, but now with the Lys843 of the two motors forced to share the same coordinates. (**A**,**B**) Lateral views across the actin filament axis (*Z* axis). (**C**) View along the direction of the filament axis. Blue lines: constrained positions of the shared Lys843s.

In rigor, in the preserved half-sarcomere architecture, the two actin−attached motors of the same myosin dimer do share the S2 attachment through Lys843, and their lever arm must be rotated relative to the unstrained conformation they have in the decorated actin. In particular, the two Lys843 are constrained to move in a circle (see Section 4) built by points that lie at the same distance (*l* = 9.56 nm) from the pivoting points of the two lever arms, here chosen as Cys707. In Figure 3, the two motors are shown with their Lys843 in the same position, in a conformation that, given the constraint, minimizes the sum of the squared distances from their unconstrained position, i.e., the position in the decorated actin filament. If the stiffness of the motor were the same in all the directions, this shared position would correspond to the conformation of the minimal elastic energy of the two-motors system. In the coordinate system used for the atomic model of acto−myosin interaction in [16], the constrained path along which the shared Lys843 can move is a circle that lies on the plane of equation:

(4) z=−0.0209·x+0.5723·y−1.3052 nm

with center (in nm) C = (−6.8896, −0.2488, −1.3008) and radius *r* = 9.015 nm.

The most obvious way to describe the distortion of the two-motor system would be the angle of rotation of the vector C-Lys843, as Lys843s describes the circumference along which they are constrained. However, in experiments aimed at determining the compliance of the elastic elements in the half−sarcomere, what is measured is the axial force (along the *z* direction, parallel to the filament axis) and the half sarcomere length change, to which the myosin motors contribute with their distortion solely along the filament axis. Additionally, if one wants the elastic response of the half−sarcomere to not be significantly affected by any confounding relaxation process, such as quick force recovery in the attached myosin motor in the actively contracting muscle that truncates the elastic response [16], or by the contamination of structure−based relaxation processes in rigor [15], the length changes imposed on the half-sarcomere should be completed within about 100 μs, and limited to an amplitude of a few (1–2) nm. For such a short amplitude, the movement along the circumference can be approximated to a straight segment.

For these reasons, for any given starting point, the best way to understand the effect of the constraint on the apparent stiffness of the two-motor system when small length steps are applied is to refer to a coordinate system with the *z* axis along the filament axis, as in the atomic coordinates from [16], and an axis orthogonal to the *z* axis and laying on the plane containing the *z* axis and the tangent to the circumference in the starting point.

This is, for example, the situation depicted in Figure 3, where the system is in a conformation that minimizes the sum of the squared distances from the unconstrained position of the two motors. In this situation, a small axial movement (along the *z* direction) produces little change in the *x* coordinate and a change in the *z* coordinate, as shown in Figure 3B, where the perspective is such that the circle constraining the Lys843 movement is seen almost exactly along the plane on which it lays. From this perspective, the circle looks like an almost straight segment, which forms an angle α ≈ 60° with the *z* axis. Thus, according to Equation (3), if the stiffness ε were the same in all the directions, then the apparent stiffness along the *z* axis would be:

(5) $$\underset{\varepsilon_{z}}{\sim }=\varepsilon \cdot \left(\tan^{2}\left(60{}^{\circ}\right)+1\right)\approx 4\cdot \varepsilon$$

As seen before, Equations (3) and (5) hold when the force (stress) applied to the two−motor system is directed along the *z* axis itself. In the sarcomeric architecture, the force on the attached myosin motors is transmitted through the *z* axis-oriented thin filament (taken here as the mechanical ground) and the S2 rod, which connects the myosin motors to the thick filament and transmits force. The distance between the axis of the thin and thick filaments is about 25 nm [18], the distance from the axis of the thin filament of the Lys843 in the conformation shown in Figure 2 and Figure 3 is about 15 nm, and the radius of the thick filament is about 8 nm [19]. With a free length of the S2 rod of about 11 nm [20] and the origin of S2 from the backbone of the thick filament facing the actin filament, these numbers imply a tilt of the S2 rod relative to the *z* axis of β ≈ 10°. Since a thick filament is surrounded by six thin filaments, azimuthally separated by 60°, the S2 azimuthal movement should be 30° at most to allow the motor to be attached to the closest thin filament, and indeed, given the relative geometry of the thick and thin filaments, this 30° azimuthal movement is the limit for the steric constraint between S2 and the backbone of the thick filament. In this situation, the angle β can be calculated as 27°. This latter value may be different, depending on the azimuthal orientation of the thin filament. However, with β ranging from 10° to 27°, if the stiffness ε were the same in all the directions, from Equation (1), the average value of $\underset{\varepsilon_{z}}{\sim }$ could be calculated as $\underset{\varepsilon_{z}}{\sim }\;\approx 2.6\cdot \varepsilon$.

It is worth remarking that the stiffness of the attached dimer in the absence of the structural constraint would be twice the stiffness of the single myosin motor, as the stiffnesses of the two motors acting in parallel combine. The factor of 2.6 represents how much the stiffness of the dimer increases in the presence of the constraint, relative to its own stiffness without constraint.

### 2.3. Limits of the Present Analysis

For the quantitative estimate of the predicted stiffness of the two-motor attached myosin dimer, we have assumed for simplicity that the two motors of the same myosin dimer share the Lys843 attachment to the S2 rod. This assumption does not take into account the steric clashes between the Regulatory Light Chains wrapped around the lever arm of the two motors that would occur in this case (not shown in Figure 2 and Figure 3). The resonance energy transfer [8] indicates that the two Lys843 may be 3.5−5 nm apart, a distance that would avoid steric clash. Recent 3D reconstructions at 1 nm resolution by electron cryo-microscopy indicated a distance of 2.8 nm for the two Lys843s [21]. This implies that, in the presence of the constraint, the increase in the head stiffness would be lower than the 2.6−fold calculated above.

We then calculated the effect of the constraint imposed by the rigor structure reported in [21], PDB file 7NEP, with a more realistic pivoting point at res721 [13], and considered the shared attachment point of the two motors halfway between the two res843′s. We have found that in this case the angle α would be 52°. Thus, from Equation (3), $\underset{\varepsilon_{z}}{\sim }\approx 2.64\cdot \varepsilon$. Taking into account the angle β of the S2 rod with the *z* axis, $\underset{\varepsilon_{z}}{\sim }$ reduces to 1.88∙ε. Thus, the relevant point of our analysis, i.e., that the two-headed attachment induces azimuthal movement of the lever arms in response to an external axial force and increases the apparent axial stiffness, is preserved.

In our analysis, we have assumed, as reported in most literature, that the two heads of a dimer bind the same actin filament. If a consistent fraction of the myosin dimers had the two heads attached to different actin filaments, this would lower the estimated increase in stiffness. Wang and colleagues have observed that only 0.6% of all heads are in a “split−head” conformation, with the two heads from one myosin molecule binding to two different adjacent thin filaments [21], a fraction so low that it would not significantly affect the results of the analysis.

A final point to be considered is whether the stiffness values ε of each of the two heads bound to actin in the rigor state are the same and close to that of the head in the contracting muscle. The rigor structure reported in [21] shows two distinct features for the lower head (closest to the Z−line) and the upper head (closest to the M−line). In particular, the heavy chain shows two different kinks between the essential light chain (ELC) and regulatory light chain (RLC) binding regions. Though this could be associated with different stiffness values between the two heads and between the heads in rigor and in the contracting muscle, the authors suggest that the angles of the kinks are likely determined by the interaction of the ELCs and RLCs, and this interaction probably stabilizes the two conformations to rigidify the lever arm, which is needed for the proper transmission of the force of the power stroke. A rigid lever arm is in agreement with the indication that the major contribution to the motor’s compliance is in the region of the converter [13], and is likely the same regardless of the two head conformations observed. It must be noted, however, that in the two heads in rigor the relative position of the motor and the ELC region is apparently the same [21], while it would be expected to be somewhat different if the two res843s were allowed to stay closer by a compliance in the converter region. A different localization of the major compliance of the motor would affect the quantitative results of our analysis.

## 3. Discussion

### 3.1. Implications for the Mechanical Measurements of the Number of Attached Motors

By our physical and geometrical considerations, we have shown that when both motors of the same myosin dimer are bound to the actin filament, the constraint imposed to their residues Lys843 to share the S2 rod attachment implies a distortion of the lever arms relative to the catalytic domains firmly attached to actin [10], and generates an axial stiffness of the pair that can be about twice that expected from the equivalent stiffness of the two single motors in parallel. This conclusion would challenge the results of experiments where the stiffness of the rigor fibers is used to determine the number of attached motors during muscle contraction [4,22,23,24]. The fraction of attached myosin motors during contraction (*f*), a fundamental parameter for the in situ definition of the stiffness and force of the motor, can be determined with fast sarcomere−level mechanics by comparing the compliance of the half-sarcomere (*C*_hs_) during contraction and in rigor [4]. *C*_hs_ results from the in−series compliances of the myofilaments (*C*_f_) and of the array of the attached motor (or cross-bridges, *C*_xb_): *C*_hs_ = *C*_f_ + *C*_xb_ [4]. The compliance *C*_xb_ is the reciprocal of the stiffness of the motor array, which is proportional to the number of motors attached to actin, *n*, in each half sarcomere. If ε_z_ is the stiffness of a single motor measured along the filament axis, then *C*_xb_ = 1/*n*∙ε_z_. From the measurements of *C*_hs_ during isometric contraction (*C*_hs0_) and in rigor (*C*_hsR_), it is possible to determine the fraction *f* of motors working in parallel in isometric contraction in each half-thick filament from the relation:

(6) $$f=\frac{C_{\mathrm{hsR}}-C_{f}}{C_{\mathrm{hs}0}-C_{f}}$$

given the assumption that the stiffness ε_z_ of the motor in rigor is the same as that during isometric contraction. However, we found that when both motors of a dimer are attached to actin, their apparent axial stiffness is influenced by the constraint of sharing the S2 rod junction, and this makes Equation (6) no longer valid. If the stiffness of a single unconstrained motor were the same, ε, along all the directions, then ε_z_ = ε for the unconstrained motors (as during isometric contraction when only one motor per dimer is attached [7]) and ε_z_ ≈ 1.9∙ε for the motors in rigor. In this case *f*, as determined by Equation (6), would be underestimated by about a factor of 2.

The comparison of *C*_hs_ in rigor and during the isometric Ca^2+^-activated contraction of demembranated fiber from rabbit psoas gives *f* = of 0.33 ± 0.05 [22] and 0.29 ± 0.08 [24], with a weighted mean of 0.32 ± 0.04. If $\underset{\varepsilon_{z}}{\sim }\approx 1.9\cdot \varepsilon$, most of the motors would be attached in the activated fiber. Indeed, if about 60% of all the motors were attached, this would mean that at least 20% of the dimers would attach to actin with both motors, and 80% with only one motor. A proportion of 20% two-headed attachment would be consistent with the 14% estimated by the cryo-electron tomography of an isometrically contracting insect flight muscle [25]. On the other hand, the fraction of motors attached under the more physiological conditions of an isometric contraction of vertebrate skeletal muscle has consistently been found to be lower than 40% [26], underpinning the lower probability of the attachment of both motors of each dimer. Structural evidence has been derived using both a spectroscopic probe on the myosin head [27] and X-ray diffraction signals [28,29]. More direct evidence that excludes the simultaneous binding of the two heads of a myosin dimer has been given by single−molecule mechanical measurements with optical tweezers, demonstrating that even at relatively low ATP, the two heads of the myosin dimer act sequentially [30], and by measurements of the mechanical performance of myosin dimers working in small array at physiological ATP, demonstrating that each head of the dimer works independently [31].

However, it cannot be ruled out that the two-headed attachment is a specific adaptation of the insect flight muscle, helping it to respond efficiently to stretch activation [25,32,33].

### 3.2. The Stiffness of the Myosin Motor Determined in Rigor and during Contraction

Early studies where EPR spectroscopy was combined with mechanical measurements in muscle fibers from rabbit psoas [26,34] found that up to 50% of motors in rigor detached upon addition of ATP analogs, with only a small reduction in the fiber stiffness. This finding was taken as evidence that only one motor in the dimer is stiff. However, it has subsequently been shown that when the large contribution (~75%) of the myofilament to the half-sarcomere compliance is taken into account, the observations are consistent with both motors having the same stiffness [22]. Indeed, it has been shown that mechanical measurements of fibers in rigor and thermodynamical considerations derived from the force–temperature relation in Ca^2+^-activated demembranated fibers converge toward the same value of ε ≈ 1.7 pN/nm for the stiffness of the myosin motor in rabbit psoas [22]. In similar experiments, convergence toward the same value for the motor stiffness in rigor and during contraction has also been found for frog [35,36]—ε ≈ 2.6 pN/nm—and for dogfish [23]—ε ≈ 2.0 pN/nm.

These results imply that the stiffness of the two motors attached in rigor is twice that of the single motor, as expected by the two stiffness values being added in parallel, and thus excluding the effect of the shared Lys843−rod attachment constraint.

Although the measurements reported above refer not to the motor itself, but to the cross-bridge, which consists of an in-series arrangement of the motor(s) with the S2 rod, both theoretical and experimental indications converge toward a high stiffness value for the S2 rod under tensile forces [8,9], and thus the stiffness of the cross-bridge does substantially coincide with the stiffness of the motor(s) [9].

### 3.3. Number and Stiffness of the Attached Myosin Motors Determined with X-ray Diffraction

The number of myosin motors attached in a [Ca^2+^]−activated demembranated fiber of rabbit psoas (pCa 4.5) has also been determined using X−ray diffraction [37], and gives an estimate of 41–43%. This figure is only 1.31 ± 0.17 times higher than the 32% determined with mechanical measurements [22,24], and rules out a 1.9-fold increase in the apparent stiffness of the motors attached in rigor.

The results of X-ray diffraction experiments on frog muscle fibers interpreted with the atomic model of the myosin motors docked to actin [16,17] have made possible to directly estimate the stiffness of the two-motor attached dimer in rigor [9,14], without the complications introduced by the myofilament’s compliance in series with that of the motors, as arise in the mechanical measurements. The changes in the X-ray signals in response to the 3 kHz oscillations applied to single muscle fibers in rigor indicate a stiffness of 2.7 ± 0.9 pN/nm [9], which, despite the high relative error, is still compatible with the results of mechanical measurements for the stiffness of the myosin motor in frog muscle, given that the stiffness of the S2 rod is much higher than that of the motor.

### 3.4. Indication for Anisotropic Stiffness of the Motor

All the above-reported measurements of the stiffness of the actin−attached myosin motor converge toward a unique value, regardless of whether a myosin dimer is attached to actin through two (rigor) or a single (active contraction) motor, though this value is different for the different myosin isoforms, which vary in relation to the species (orthologous isoforms) or muscle types in the same species (i.e., fast or slow, paralogous isoforms). These results indicate that $\underset{\varepsilon_{z}}{\sim }\approx 2.6\cdot \varepsilon$, or even $\underset{\varepsilon_{z}}{\sim }\approx 1.9\cdot \varepsilon$, derived under the assumption that the stiffness of the attached motor dimer is isotropic ($\varepsilon =\varepsilon_{x}=\varepsilon_{z}$), does not fit with the experimental data. Our analysis of the apparent stiffness of the constrained two-motor attachment may be reconciled with the experimental results if, considering Equation (2), $\varepsilon_{x}$ (the lateral stiffness of the attached myosin motor) is much smaller than $\varepsilon_{z}$ (its axial stiffness).

An indication of the anisotropy of the motor stiffness arises from the results of the measurements reported by Billington and colleagues [38]. In that paper, by applying negative stain electron microscopy and image processing to the free myosin fragment S1, the authors estimated an apparent stiffness of the motor of 0.37 pN/nm. This value is about 3 to 7 times lower than the values of 1.2–2.7 pN/nm reported in the literature, derived from experiments wherein the stiffness was estimated for the motor in situ, as discussed in the previous sections. Billington and co−workers suggest that the discrepancy may be due to the allosteric stiffening of the motor upon binding to actin. Though a different stiffness for the myosin motor depending on its nucleotide state has been recently indicated [39], the results of our analysis compared with the experimental results reported in Section 3.2 and Section 3.3 suggest a different interpretation.

Our interpretation is that the stiffness of the motor is anisotropic, and it is higher along the *z* axis (along the thick filament) than along the orthogonal *x* direction. Since the motors in the experiments reported in [38] are free and appear randomly oriented along their longer axis, the distribution of angles between the lever arm and the catalytic domain is also random with respect to the direction of the higher $\varepsilon_{z}$ and the lower $\varepsilon_{x}$ stiffness. In this condition, the apparent average stiffness $\hat{\varepsilon }$ can be derived from the mean square of the displacement of the tip of the lever arm, $\langle r^{2}\rangle$, through the equipartition of the energy (with two degrees of freedom associated to the two orthogonal directions): $\frac{1}{2}\hat{\varepsilon }\langle r^{2}\rangle =k_{B}T$. Comparing $\frac{1}{2}\varepsilon_{x}\langle x^{2}\rangle =\frac{1}{2}k_{B}T$ and $\frac{1}{2}\varepsilon_{z}\langle z^{2}\rangle =\frac{1}{2}k_{B}T$, and considering that $\langle r^{2}\rangle =\langle x^{2}\rangle +\langle z^{2}\rangle$, one gets $\hat{\varepsilon }=2\cdot \frac{\varepsilon_{x}\cdot \varepsilon_{z}}{\varepsilon_{x}+\varepsilon_{z}}$ or $\varepsilon_{x}=\frac{\hat{\varepsilon }\cdot \varepsilon_{z}}{2\varepsilon_{z}-\hat{\varepsilon }}$.

From the latter relation, with $\hat{\varepsilon }=$ 0.37 pN/nm and $\varepsilon_{z}=$ 1.2–2.7 pN/nm, $\varepsilon_{x}\approx$ 0.2 pN/nm. Thus, the lateral stiffness of the actin-attached myosin motor in situ ranges from 1/6 to 1/13 (average 1/8) of the axial stiffness. With these numbers and α = 60°, Equation (2) gives on average $\underset{\varepsilon_{z}}{\sim }$ = 1.38 ε_z_, i.e., the axial stiffness of the motor in an attached dimer is about 40% higher than that of a single attached motor. With this estimate for $\underset{\varepsilon_{z}}{\sim }$, the number of actin-attached motors per half−thick filament during isometric contraction obtained from mechanical measurements using Equation (6) is underestimated by ca 30%. This would account for the difference reported above between the fraction of attached motors in rabbit psoas as determined by X-ray measurements (≈ 41–43%, not depending on stiffness measurements) and by mechanical measurements (32%). Indeed with the angle α = 52°, as estimated from the structural model of Wang and coworkers ([21]; PDB 7NEP), we obtain $\underset{\varepsilon_{z}}{\sim }$ = 1.20 ε_z_ or less. In this case, the difference in the fraction of attached motors given by mechanical and X-ray measurements cannot be explained only with the higher apparent stiffness of the motors in rigor. We conclude then that the stiffness of the myosin motor is anisotropic, with the lateral stiffness about or less than one order of magnitude the axial stiffness.

Whatever its exact value, the fact that the azimuthal stiffness is one order of magnitude lower than the axial stiffness explains why the stiffness values of the motor obtained with mechanical measurements and with thermodynamical consideration converge toward a unique value, as discussed in Section 3.2. Thus, the estimate of the fraction of attached motors obtained by comparing the compliance of the half−sarcomere (*C*_hs_) during contraction and in rigor is largely justified.

### 3.5. Energetic Considerations

The anisotropy of the stiffness of the cross−bridges has also been suggested by Koubassova and colleagues [40], applying the principle of minimal elastic distortion energy to actin labeling to simulate the X-ray patterns observed in rigorized muscle fibers taken from rabbit psoas. There, the stiffness considered was that of the whole cross−bridge, i.e., the series of S1 and S2 fragments. In the present study, we have determined that the anisotropy is inside the S1 fragment, the myosin motor itself.

Since two consecutive monomers on the same strand of the actin helix are azimuthally rotated by ca 30° relative to each other, the low azimuthal stiffness of the myosin motor can be seen as functional to facilitate the actin attachment of the catalytic domain. The estimated 0.2 pN/nm lateral stiffness of the myosin motor indicates that the root mean square displacement of the tip of the lever arm relative to the hinge with the catalytic domain has a value of about 4.5 nm (again, from the equipartition of energy). With a length of the lever arm of about 9 nm, this means a root mean square of the azimuthal angle between lever arm and catalytic domain of about 26°, which fits well with the azimuthal displacement of consecutive actin monomers along the helical strand, allowing the motor to attach to one or the next without difficulty, whichever better matches its axial position, and it accounts for the second head attachment on the next Z-ward actin monomer in response to a sudden increase in load, as suggested by the mechanical and X-ray diffraction experiments [33].

To conclude, the high stiffness of the myosin motor along the axial direction is able to transmit high force resulting from the structural changes in the motor associated with the working stroke. The much lower stiffness in the azimuthal direction facilitates the motor’s attachment to the actin monomers, which expose their attachment sites for myosin with a large azimuthal range.

## 4. Materials and Methods

To characterize the apparent stiffness in one given direction when the structure constrains the movement of the tip of one elastic element, we have first built a mathematical representation of a 2D physical model (Figure A1). The elastic element is represented with two orthogonal springs with stiffness ε_x_ and ε_z,_ corresponding to the stiffness of the element along the *x* and *z* directions, respectively, connected to the *z* and *x* axes, which act as the mechanical ground, and together form the tip of the elastic element. The tip of the elastic element, then, is constrained to move in a direction that forms an angle α with the *z* axis. We have calculated, using the diagram of the forces, the relation between the force F (the stress) applied to the tip of the elastic element and the corresponding elongation (strain) (Appendix A).

To evaluate the effects of the structural constraints of the two motor attachments in rigor on their compound stiffness, we have built a structural model based on the crystallographic coordinates of the acto−myosin complex described in [16] (PDB files available as Supplemental Material in [16]). In that model, the actin filament shows an axial repeat of 2.75 nm and a 28/13 symmetry, i.e., there are 28 actin monomers for every 13 turns of the helix, and the two next monomers are rotated relative to the filament axis by 360°∙13/28 = 167.14°. Thus, two consecutive monomers on one strand of the double-stranded helix are rotated by an angle θ = (167.14°∙2=) 334.28° (or ca −26°), and axially shifted by 5.50 nm.

In rigor, when all the myosin motors are assumed to be attached to actin, the two motors of the same dimer are considered attached to two consecutive actin monomers on the same strand of the helix. We have thus applied the same axial shift and azimuthal rotation to the coordinates of the myosin motor, in order to represent the two motors attached, with the same conformation, on the two consecutive actin monomers, namely:

(7) $$\{\begin{array}{c} x2=x1\cdot \cos\left(\theta \right)-y1\cdot \sin\left(\theta \right)\\ y2=x1\cdot \sin\left(\theta \right)+y1\cdot \cos\left(\theta \right)\\ z2=z1+5.5 \end{array}$$

where (x1, y1, z1) is the set of coordinates for one motor (as in the original PDB file), and (x2, y2, z2) are the coordinates for the other (partner) motor.

The lever arm of each motor is allowed to rotate as a rigid body around a pivoting point in the converter domain, which has been chosen as Cys707 [11,12,41].

Thus, the tips of the lever arms of the two motors (Lys843) may move on the surface of a sphere, centered at their Cys707 coordinates with a radius corresponding to the length of the lever arm *l* = 9.56 nm, taken as the Cys707–Lys843 distance.

When the two Lys843 are constrained to be attached to the S2 rod sharing their attachment point, they are also constrained to move along a circle, i.e., at the intersection of the surfaces of the two spheres. Thus, this circle represents the constraint of the distortion of the system constituted by the two actin-attached motors of the same myosin dimer.

## Figures and Tables

**Figure A2:**
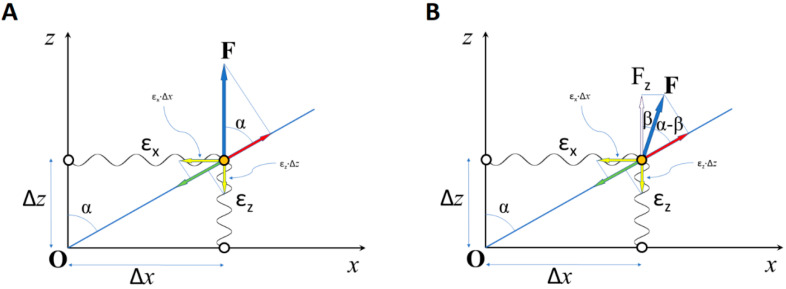
Schematic 2D representation of an elastic element with different stiffness values of ε_x_ and ε_z_ along the *x* and *z* directions, respectively. The blue line at angle α with the *z* axis represents the structural constraint of the movement of the tip of the element (orange circle) when a force **F** (blue arrow) is applied. Red arrows: component of the force F along the constraint; green arrow: restoring forces along the constraint, at equilibrium; yellow arrows: restoring forces along the *x* and *z* axis; white arrow: projection of F along the *z* direction. (**A**) Force **F** parallel to the *z* axis. (**B**) Force **F** at a generic angle β with the *z* axis.

## Data Availability

Not applicable.

## Appendix A

As in Figure A1, we have represented the elastic element in 2D as an orange circle connected to two orthogonal springs with stiffness ε_x_ and ε_z_. The white circles at the other ends of ε_x_ and ε_z_ springs that can slide frictionlessly along the *z* and *x* axis, respectively, which represents the mechanical grounds. We set the rest length of each spring to be zero, i.e., without any external force, the equilibrium point for the system is with the orange circle in **O**, the origin of the axes. If an external force **F** is applied to the orange circle, a new equilibrium is found when the restoring forces of the two springs act together, so that their vectorial sum balances the external force. In particular, the spring ε_x_ must be extended by Δ*x* so that ε_x_∙Δ*x* = F_x_, the component of the force **F** along the *x* direction, and similarly, we derive ε_z_∙Δ*z* = F_z_ for the *z* direction.

We now look at Figure A2A, where the orange circle is constrained to move, frictionlessly, along the fixed blue bar that originates from **O** and forms an angle α with the *y* axis. If an external force **F** directed along the *y* direction (blue arrow) is applied to the orange circle, the component of the force normal to the blue bar will be balanced by both the restoring forces of the springs along that direction and the force applied by the bar, while the component of the force along the bar, F∙cos(α) (red arrow), can be balanced only by the restoring forces of the two springs in that direction, the bar being unable to exert force along its axis. The restoring forces of the two springs are represented by the yellow arrows, and their components along the bar are added to give ε_x_∙Δ*x*∙sin(α)+ ε_z_∙Δ*z*∙cos(α) (green bar).

At the equilibrium

(A1) $$F\cdot \cos(\alpha )=\varepsilon_{x}\cdot \Delta x\cdot \sin(\alpha )+\varepsilon_{z}\cdot \Delta z\cdot \cos(\alpha )$$

the constraint for the orange circle to move along the bar implies Δ*x*/Δ*z* = tan(α), and thus:

(A2) $$F=\varepsilon_{x}\cdot \Delta z\cdot \tan^{2}(\alpha )+\varepsilon_{z}\cdot \Delta z=\Delta z\cdot (\varepsilon_{x}\cdot \tan^{2}(\alpha )+\varepsilon_{z})$$

Equation (A2) indicates that when a force is applied along the *z* direction, the constraint forcing the tip of the elastic structure (the orange circle) to move along the bar results in an elastic response with a stiffness

(A3) $$\underset{\varepsilon_{z}}{\sim }=\varepsilon_{x}\cdot \tan^{2}(\alpha )+\varepsilon_{z}\;$$

We call $\underset{\varepsilon_{z}}{\sim }$ the *apparent stiffness* along the *z* direction. Similarly, the compound stiffness along the *x* direction, i.e., the ratio between an external force applied along the *x* direction and the strain Δ*x* at the equilibrium, will be

(A4) $$\underset{\varepsilon_{z}}{\sim }=\varepsilon_{x}+\varepsilon_{z}\cdot {\mathrm{cotan}}^{2}(\alpha )$$

In the general case that force **F** is not oriented along *z* but forms an angle β with *z* (Figure A2B), the component of **F** along the *z* direction is F∙cos(β) and along the bar it is F∙cos(α-β). In this case, the compound stiffness along *z* is

(A5) $$\underset{\varepsilon_{z}}{\sim }=\cos\left(\beta \right)\cdot \left\{\varepsilon_{x}\cdot \frac{\sin^{2}\left(\alpha \right)}{\cos\left(\alpha \right)\cdot \cos\left(\alpha -\;\beta \right)}+{\;\varepsilon }_{z}\cdot \frac{\cos\left(\alpha \right)}{\cos\left(\alpha -\;\beta \right)}\right\}$$

and along *x* it is

(A6) $$\underset{\varepsilon_{z}}{\sim }=\sin\left(\beta \right)\cdot \left\{\varepsilon_{x}\cdot \frac{\sin\left(\alpha \right)}{\cos\left(\alpha -\;\beta \right)}+{\;\varepsilon }_{z}\cdot \frac{\cos^{2}\left(\alpha \right)}{\sin\left(\alpha \right)\cdot \cos\left(\alpha -\;\beta \right)}\right\}$$
